# Effect of grain dissolution on sloping ground

**DOI:** 10.1038/s41598-022-26620-1

**Published:** 2022-12-23

**Authors:** Minsu Cha, J. Carlos Santamarina

**Affiliations:** 1 Department of Civil Engineering, College of Ocean Sciences, Jeju National University, Jeju-Si, Jeju Special Self-Governing Province 63243 Republic of Korea; 2 Earth Science and Engineering, King Abdullah University of Science and Technology (KAUST), Thuwal, 23955-6900 Saudi Arabia

**Keywords:** Natural hazards, Geomorphology, Civil engineering

## Abstract

The static and dynamic stability of natural or constructed slopes can be affected by dissolution or dissolution-like phenomena. Their underlying mechanisms, however, remain unclear. New experimental results and discrete element simulations provide particle-level and macroscale information on the consequences of mineral dissolution on slope behavior. At the microscale, load-carrying grain arches develop around dissolving particles, the porosity increases, and contact force chains evolve to form a honeycomb topology. At the macroscale, while vertical settlements are the prevailing deformation pattern, lateral granular movements that create mass wasting are prominent in sloping ground, even under the quasi-static granular loss. Horizontal grain displacement is maximum at the surface and decreases linearly with the distance from the slope surface to become zero at the bottom boundaries, much like vertical granular displacement along the depth. Sediments with smaller friction angles and steeper slopes experience greater displacement, both vertically and horizontally. Slopes become flatter after dissolution, with the reduction in slope angle directly related to the loss in ground elevation, ΔH/H_o_. Yet, because of the porous fabric that results from dissolution, vertical shortening is less than the upper bound, estimated from the loss in the solid mass fraction, ΔH/H_o_≈SF. Under water-saturated conditions, the post-dissolution fabric may lead to sudden undrained shear and slope slide.

## Introduction

Dissolution and reprecipitation are prevalent and persistent diagenetic processes. The time scale for chemical processes is typically quite long and the “inert assumption of soils” applies to many engineering applications. However, dissolution and precipitation can also take place within relatively short time scales in advective regimes such as infiltration-induced carbonate dissolution and when systems are taken far from equilibrium in young systems such as dam foundations, mine tailings, fly ash, volcanic ash, and CO_2_ injection^1–13^.

Waste is particularly vulnerable to dissolution and degradation because its components are suddenly exposed to new environmental conditions outside equilibrium. Heavy metals and acid drainage from mine tailings and fly ash ponds are early signs of ongoing dissolution processes^14,15^. Likewise, some components in fly ash, including CaO and CaSO_4_, are highly soluble^16,17^. The dissolution of coal ash grains contributes to instability and collapse^18^; on the other hand, new minerals such as zeolite and phillipsite can precipitate^19,20^. Dissolution and reprecipitation lead to porous yet cemented sediments that are brittle and contractive and are vulnerable to liquefaction once perturbed. The potential for slope instability due to solid phase loss could affect waste disposal (including organic waste, mine tailings, and coal ash).

Many natural ground movements involve some form of solid phase loss, albeit not necessarily mineral dissolution. Repetitive diurnal or seasonal freeze–thaw that involves the thawing of ice needles and segregated ice cause a downslope ratcheting movement, termed solifluction^21–24^. The failure of the Carsington Dam occurred along a pre-existing solifluction shear plane within the foundation layer^25,26^. In recent decades, diminishing permafrost in high mountains and polar regions has increasingly caused landslides^27–30^ and contributed to the destabilization of rock structures^31,32^.

Similarly, gas hydrate dissociation is one of the major causes of large-scale submarine landslides^33–37^, seen in the Storegga Slide^38,39^ and the Trænadjupet Slide^40,41^. Hydrate dissociation not only involves solid mass loss but also accompanies gas generation and expansion, which results in marked excess pore pressure generation and effective stress loss^33,42^. Gas hydrate dissociation can be naturally triggered by sea-level fluctuations^43^ and rising ocean temperatures^44^, and is unavoidable in the vicinity of platform foundations^34,45^. In addition, dissolution may be purposely caused for gas production^46^.

As described above, many studies have attributed slope movement in the field to dissolution or dissolution-like phenomena. To date, however, no study has closely examined the effects of the mechanisms of dissolution on the behavior of sloping ground. Therefore, the purpose of this research is to explore the stability and deformation of sloping ground due to solid phase loss such as mineral dissolution. In particular, we aim to understand mass wasting characteristics during dissolution in dry, drained conditions using micro- and macro-scale information gathered from discrete element simulations and any emergent behavior when dissolution occurs underwater using a laboratory model test.

The research approach combines a high-caliber 1 g laboratory experiment and a series of discrete element simulations. Note that the two approaches are not designed to match the conditions of each method but to complement and harness their respective advantages. For example, the experiment uses saturated specimens while the DEM employs dry specimens.

## Experimental study

We designed and conducted a 1 g laboratory experiment to explore the consequences of dissolution in slopes under water-saturated conditions, which is a common field condition during dissolution.

### Device and specimen

**Figure 1:**
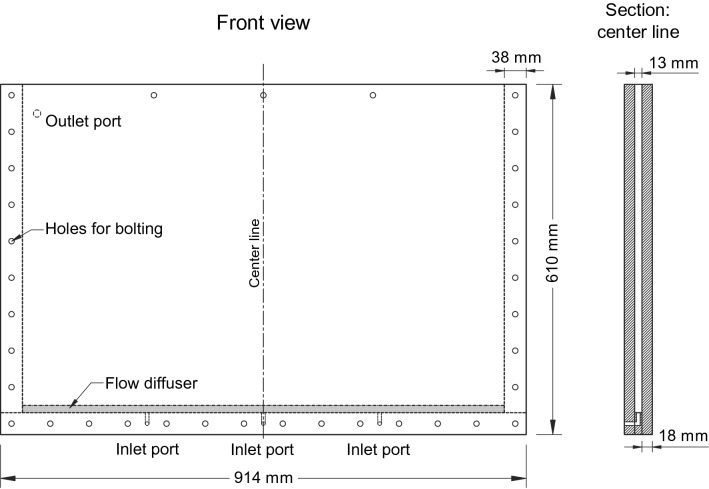
Transparent two-dimensional tank used to explore the effects of dissolution on slope stability.

The experimental study used a thin tank made of two parallel, transparent acrylic plates (width 18 mm, separated by a 13 mm gap; Fig. 1). The thick acrylic walls were 914 mm × 610 mm and firmly fixed to spacers at the four edges of the tank to minimize the horizontal deflection of the walls, which results from the sediment weight. Inlet ports at the bottom were covered with a flow diffuser that ran along the full base beneath the sediment, which enabled a true 1D flow and promoted uniform dissolution (Fig. 1).

The results presented herein correspond to a sediment made of 90% sand (insoluble grains: D_50_ = 0.16 mm, G_s_ = 2.65, subround, poorly graded, a global friction angle of 38°) and 10% salt (soluble grains: D_50_ = 0.3 mm, G_s_ = 2.165, cubical, poorly graded) by weight, creating a mixture with a soluble fraction (SF) of 10%. Sand and salt were dry-mixed and then pluviated into the tank, forming the slope, with an initial angle of 30°. As the specific gravities of insoluble (G_U_ = 2.65) and dissolvable grains (G_D_ = 2.165) differ, we used their weighted average to calculate the initial global porosity:

1 $$\mathrm{n}=1-\frac{{\rho }_{dry}}{{\rho }_{w}}\frac{\left(1-m\right){G}_{D}+m{G}_{U}}{{G}_{U}{G}_{D}}$$

where *m* = mass fraction of dissolvable grains; and densities ρ_w_ and ρ_dry_ correspond to water and the dry mixture, respectively. The initial porosity measurement n_0_ is 0.412 (medium loose).

### Test procedure and conditions

The model was slowly flooded with a saturated NaCl brine solution permeated from the bottom (duration: 5 h); brine prevented the dissolution of the salt mixed in with the sand. The gradual dissolution was controlled by progressively lowering the salt concentration in the permeated fluid (duration: 3 days). These slow and progressive changes in salt concentration over the long period ensured all soluble particles decreased in size at the same time (a “homogeneous dissolution” as in Cha and Santamarina^47^). The low reaction rate (low Damkohler number) guaranteed uniform dissolution and avoided preferential dissolution modes such as localized dissolution and face dissolution initiated from the inlet boundary. In addition, a very low hydraulic gradient (i < 0.1) was maintained for the saturation and flow so that it created negligible uplift pressure on the sediment particles. A camera recorded the slope deformation by high-resolution time-lapse imaging with a five-minute time interval. The experimental setup was isolated on a heavy, vibration-free table during dissolution.

While it is impossible to simulate the time scale of dissolution in the field, we ensured that the laboratory dissolution was very gradual, as demonstrated by the long and uniform dissolution. Consequently, the system behaved quasi-statically.

Previous research has used the inertial number (I) to quantify the dynamic effects in particulate discrete element simulations^48^ and experimental analysis^49^. The calculated inertial number was ~ 10^−8^ for the particles on the surface, which is well within quasi-static criteria (I < 10^−3^).

### Experimental results

**Figure 2:**
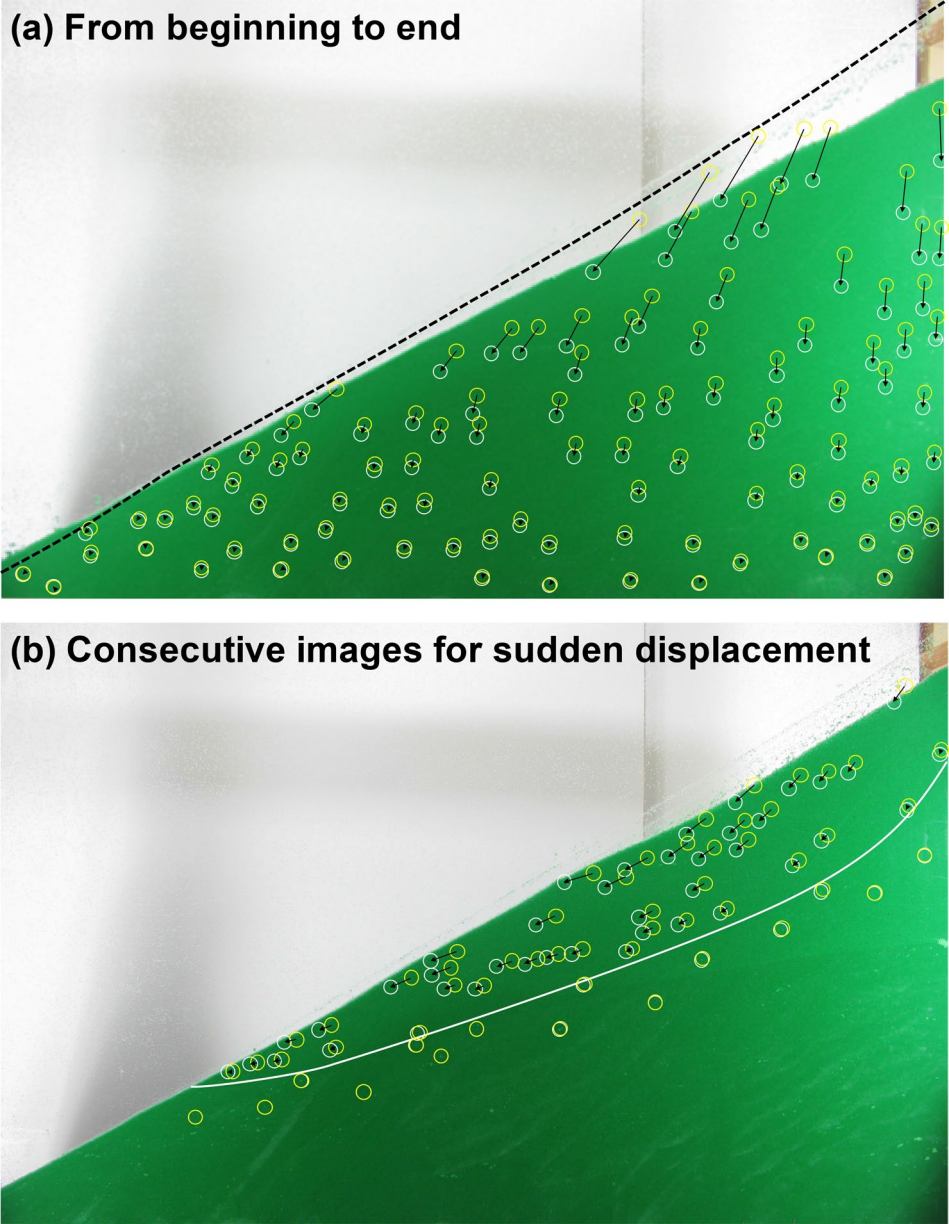
Experimental results. Displacement vectors at visually recognizable points: (**a**) From the beginning to the end of dissolution. Note: The dotted line indicates the original slope surface; (**b**) Sudden shear failure observed during a 5-min photo-interval. This test corresponds to a specimen mixed with a mass fraction SF = 10% of dissolvable particles. Note: Displacement vectors are shown at the same scale in both (**a**) and (**b**).

Digital image correlations failed to provide adequate displacement fields—from the start to the end—due to a lack of contrast in the sediment. Instead, we manually traced displacements of clearly visible and persistent impurities detectable in high-resolution images. The results in Fig. 2a show the displacement vectors from the beginning to the end of dissolution, which demonstrate that vertical displacements prevail, the magnitude of vertical displacements increases almost linearly with elevation, and horizontal displacements become increasingly prominent towards the slope surface. An areal analysis of the specimen shows that the total volume decreases by 7.5%, with the dissolved fraction of 12% by solid volume (10% by weight), which provides the calculation of the increase in porosity from the initial porosity n_0_ = 0.412 to final porosity n_f_ = 0.440.

Notably, we detected a sudden displacement when all the recorded images were played as a movie sequence. Figure 2b presents the two consecutive images that highlight this sudden displacement. Digital image correlation did capture the sudden movement between these consecutive images; see Fig. S1 in the Supplemental Data. The displacement vectors defined a shallow failure surface. The maximum possible duration for this local failure is the time interval between photographs Δt = 5 min (although the fact that the localization is solely contained within the two consecutive images indicates the actual duration could be arbitrarily much shorter). Given a drainage length similar to the slide depth, d = 10 cm, the coefficient of consolidation for drained conditions should be higher than c_v_ > d^2^/Δt = 0.3 cm^2^/s, which corresponds to fine sands. These results suggest that this local failure took place under undrained conditions.

**Figure 3:**
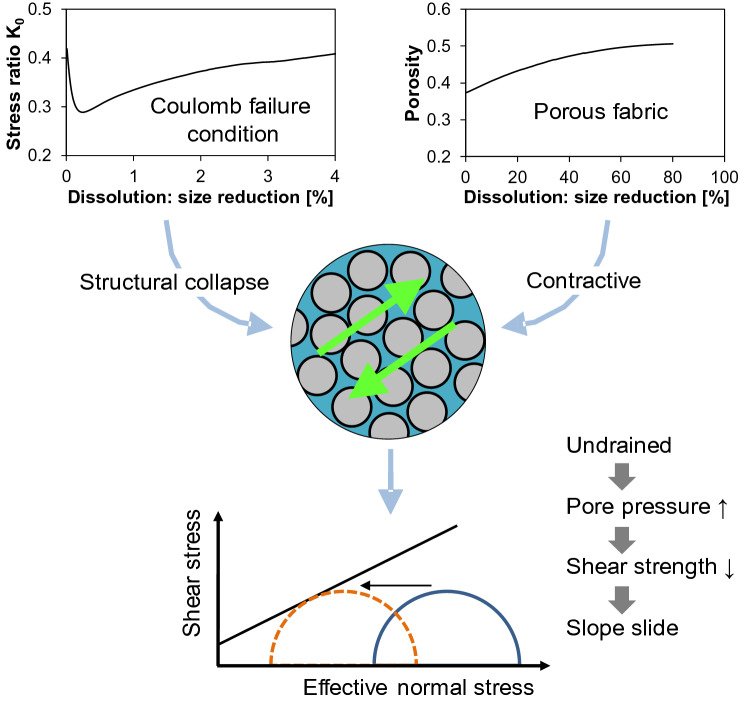
Sequence of conditions leading to the slide. The stress ratio K_0_ and porosity are from the three-dimensional discrete element simulations in Cha and Santamarina^47^.

A possible mechanism leading to the slide could involve the following sequence of events, as also illustrated in Fig. 3. Granular dissolution results in increased porosity, as shown in this experiment and other work, and the sediment becomes contractive^47^. At the same time, internal stress states approach the Coulomb failure condition, i.e., K_a_^50^. Therefore, the active failure condition initiated the structural collapse of the porous granular skeleton. This sudden collapse combined with the highly contractive tendency of the post-dissolution sediments^47^ can lead to the temporal generation of excess pore pressure, as described in the above paragraph, and a decrease in the effective stress, which lowered shear strength and resulted in the slope failure/slide.

We used low-friction, scratch-free acrylic plates for the lateral walls. Nevertheless, the boundaries may create friction between the particles and lateral walls and could lessen granular movement at the boundaries to some degree. First, during dissolution, friction may reduce settlement and then once shear localization initiates, it will potentially reduce the amount of movement. Indeed, our shear slide occurred over a short distance. The fact that the localization occurred in spite of the boundary friction may indicate a more substantial slide in a 3D experiment with a large lateral dimension.

## Discrete element simulations

The consequences of dissolution on sloping ground were studied further using two-dimensional (2D) discrete element method (DEM) simulations via Particle Flow Code (PFC) 2D by Itasca. Unlike the experiments, the DEM simulations were dry or drained (no fluid module). These conditions still have relevance in dissolution under unsaturated conditions (e.g., by repeated rainwater infiltrations)^51,52^, and solifluction in partially saturated ground, or even saturated slopes of coarse-grained sediments that do not experience localization during dissolution. While not emulating the exact conditions, the simulations and experiments gather complementary information.

### Simulation environment and geometry

**Figure 4:**
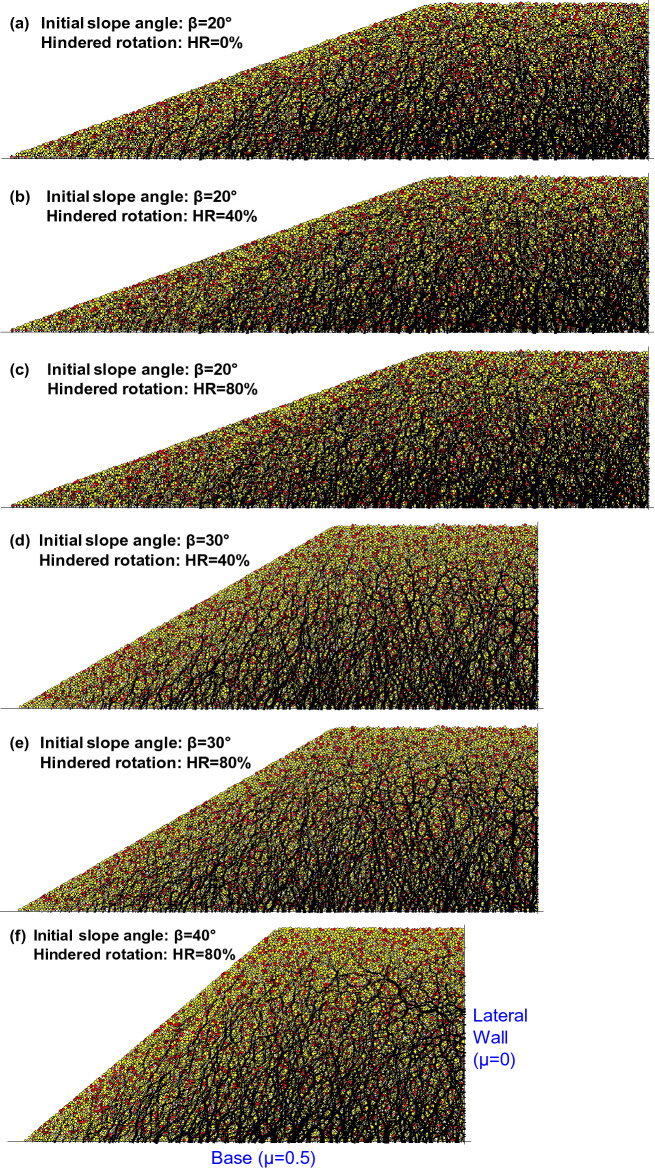
Specimen preparation and contact force chains before dissolution – All cases are shown at the same force scale. Soluble particles are shown in red (SF = 25%). Note the number of particles are the same for all simulations (10,567).

Table 1 shows the basic simulation environment and material properties. The 2D simulations used the linear contact model with normal stiffness k_n_ = 10^8^ N/m and shear stiffness k_s_ = 10^8^ N/m. As noted above, conditions were dry/drained, and there was no side friction against the front or back walls. Additionally, there is no friction between the sediment and the vertical lateral walls, which allowed the 1D settlement of the level ground portion subjected to uniform dissolution. The friction coefficient between the sediment and the lower boundary (non-dissolving base material) was 0.5 (Fig. 4f). The stiffnesses of the contact model and the inter-particle friction were selected based on the literature and the software manual^53–56^. The sediment consisted of disks that were placed at random locations within the model geometry and allowed to grow to their final target size (i.e., uniform size distribution with R_min_ = 0.4 mm and R_max_ = 0.6 mm).

**Table 1:** Two-dimensional discrete element simulation environment.

	Properties	Values
Particles (disks)	Particle size distribution	Uniform size distribution (R_min_ = 0.4 mm, R_max_ = 0.6 mm)
	Number of particles	10,567
	Mass density of particles	2650 kg/m^3^
	Linear contact model	Normal stiffness k_n_ = 10^8^ N/m
		Shear stiffness k_s_ = 10^8^ N/m
	Inter-particle friction	0.5
	Fraction of soluble particles	25%
	Hindered rotation, HR	0%, 40% and 80%
Boundary conditions	Initial slope angle	20°, 30° and 40°
	Particle-to-wall friction	0.5 for the base 0 for the wall

Grain angularity and interlocking, which imply rotational resistance, can be numerically implemented by imposing rolling resistance^57–60^ or by the introduction of non-spherical particles and granular clusters^61–64^. Hindered particle rotation is a computationally efficient approach that accounts for grain angularity^65,66^. Numerical results are physically inconsistent when all particles have hindered rotation; in this study, we hindered the rotation of a preselected percentage of randomly located particles, that is, hinder rotation (HR) of 0%, 40%, and 80%, to simulate different levels of granular interlocking or macroscopic friction^67^. For a discussion and calibration of this approach with the experimental results of Shin and Santamarina^50^, see Cha and Santamarina^47^. Although this approach did mimic the macroscopic trends and global-scale micromechanical parameters, it created more unnatural behaviors than those from computationally expensive techniques that use realistic particle shapes; in particular, we observed excessive, localized dilation around rotationally hindered particles.

Simulations used three initial slope angles: β = 20°, 30°, and 40° (Fig. 4). The numerically measured angle of repose, Φ_r_, increased with the percentage of particles with hindered rotation: Φ_r_ = 22° for HR = 0%, Φ_r_ = 35° for HR = 40%, and Φ_r_ = 48° for HR = 80%^67^. Therefore, only the slope with β_o_ = 20° was stable when HR = 0%; the slopes with β_o_ = 20° and 30° were stable for HR = 40%; and the three slopes with β_o_ = 20°, 30°, and 40° were stable when HR = 80%. The specimen dimensions for the β = 20° cases are a base length of 284 mm and height of 72 mm.

### Dissolution simulation

Soluble particles were randomly selected and, thus, homogeneously distributed in the preformed granular packing (soluble particles shown in red; Fig. 4). The mass fraction of soluble particles (SF) accounted for SF = 25% of all particles. Note soluble fractions in a given sediment that contains water-soluble minerals such as carbonate and evaporite can vary greatly^68,69^.

Smooth and gradual size reduction prevented numerical instability and dynamic effects. The dissolution of soluble particles was performed by gradually and simultaneously reducing the radius of all the soluble particles at the same rate^70^. In particular, the gradual size reduction involved numerous steps of minute size reduction (specifically, a radius reduction of 1/50,000 times the initial radius in each step), with each step followed by a full equilibrium stage^47,71^. In addition, the ratio of the mean unbalanced force to the mean contact force was always smaller than 0.001, which ensured stable conditions throughout the dissolution process.

The inertial number I is the ratio between the time for a given displacement when accelerated by the stress-dependent skeletal forces σ'd^2^ and the time for the same displacement given an imposed strain rate $\dot{\upgamma }$^48,72^:

2 $$I = \frac{{\dot{\gamma }d}}{{\sqrt {{\raise0.7ex\hbox{${\sigma^{\prime } }$} \!\mathord{\left/ {\vphantom {{\sigma^{\prime } } \rho }}\right.\kern-0pt} \!\lower0.7ex\hbox{$\rho $}}} }}$$

For particles of diameter d = 1 mm, grain density ρ = 2650 kg/m^3^, average effective stress σ' = 0.02 kPa of surface particles (the most critical case), and particle shrinking rate $\dot{\upgamma }$ = 0.017/s, the computed inertial number I≈2 × 10^−4^ is within the quasi-static criterion I < 10^−3^ for strain rate independent frictional resistance. For the global movement, applying a slope movement rate $\dot{\upgamma }$ = 0.004/s and an effective stress 0.6 kPa (at mid depth), the calculated I is 8 × 10^−6^.

Macroscale parameters stabilized when the sizes of dissolvable particles decreased to 10% of the initial sizes. We ended our simulations when the particle sizes reduced to 1% of their initial sizes. The time step was set to $\Delta \mathrm{t}=0.6\sqrt{\mathrm{m}/\mathrm{K}}$ , where *m* is the particle mass and *K* the particle stiffness; the time step changed as the grain mass *m* diminished during the simulations. The physical time for the dissolution simulations was about 2 min while the computation time exceeded several weeks. Observations from the simulation results follow.

### Contact force and internal fabric

**Figure 5:**
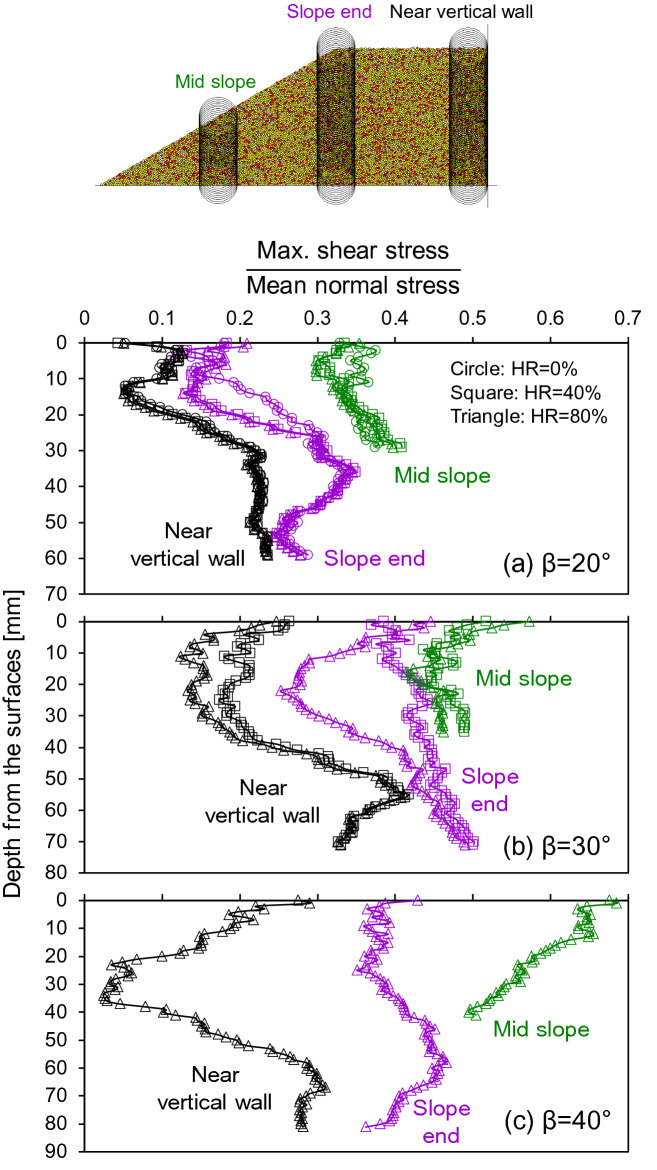
The distribution of maximum shear stress divided by the mean normal stress in the mid slopes, slope ends and near the vertical walls. Note each data point is the average value within the measurement circles located as shown.

Contact forces form characteristic “chains” in granular materials. Force chains are evenly distributed before dissolution in our simulations and have a preferentially vertical orientation in agreement with the typical stress ratio K_0_ ≈ 0.45 under normal compaction, yet the principal direction of force chains deviate from the vertical near the slope (Fig. 4). Figure 5 shows that higher stress anisotropy exists in the sloping ground (from the surface to the base) in its initial condition. The larger slope angle creates higher values of maximum shear stresses divided by the mean normal stresses.

**Figure 6:**
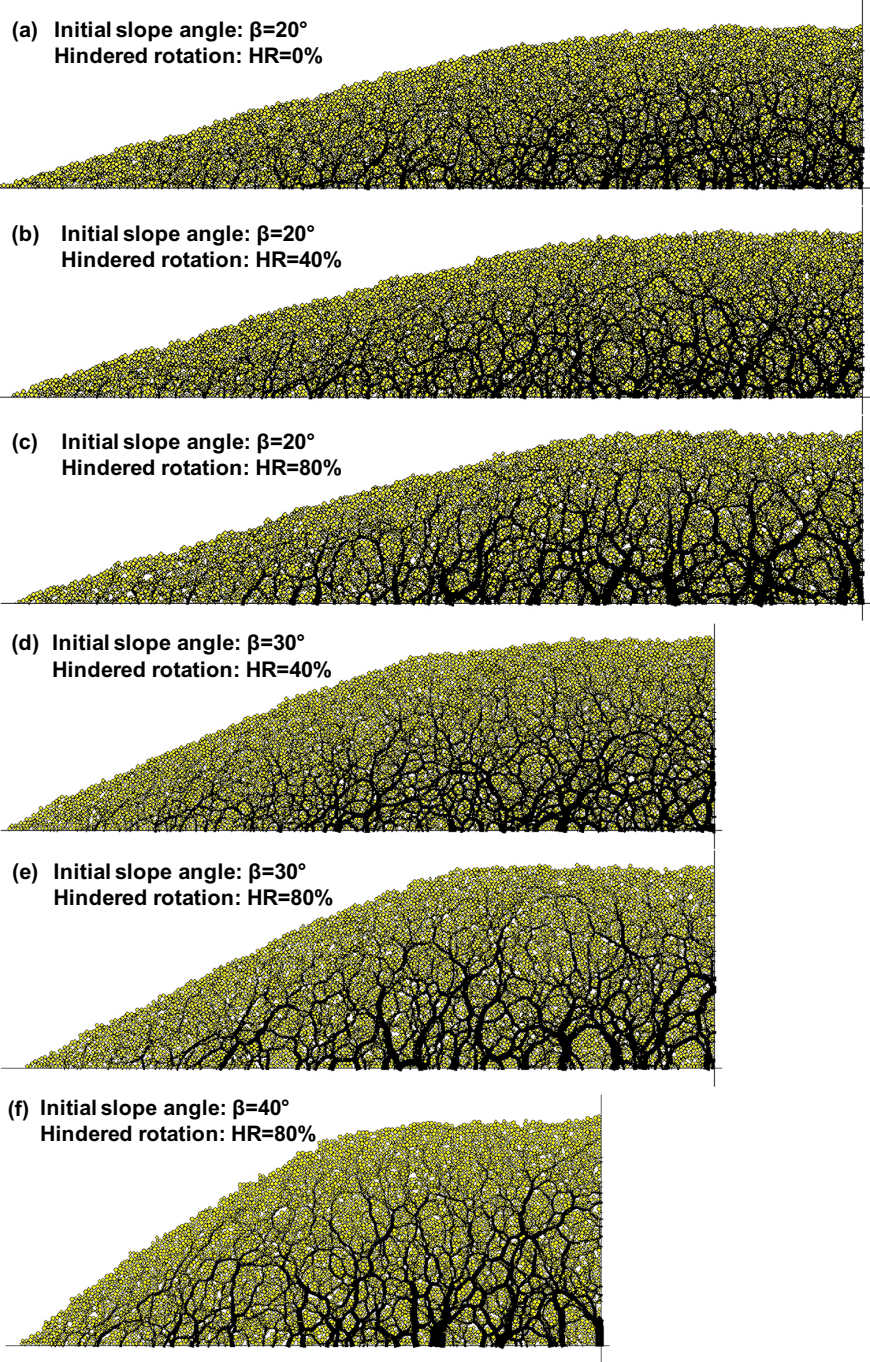
Contact force chains at the end of dissolution – All cases are shown at the same force scale. Note: Compare these results to those shown in Fig. 5.

Force redistribution starts after the dissolvable particles begin to contract; forces initially carried by dissolvable grains transfer to neighboring grains during dissolution (see the video—Supplementary Data). Preponderant interparticle force chains form arches around developing voids in a honeycomb-like fabric (Fig. 6). The honeycomb-shaped network of force chains was more prominent in sediments with more granular interlocking, that is, packings with a higher fraction of rotationally hindered particles, HR (Fig. 6). The coordination number decreased for all cases: from an initial value of 3.62 (all cases), to 2.38 (no hindered rotation), 2.28 (HR = 40%) and 2.09 (HR = 80%) at the end of dissolution. The state of stress became more stable after dissolution, especially for the mid slope.

### Porosity and pores

The final overall porosity increased in all cases: from an initial value of n_o_ = 0.155 (Dense; all cases), to n = 0.205 (no hindered rotation), n = 0.216 (HR = 40%) and n = 0.243 (HR = 80%) at the end of dissolution, similar to the experimental results. Complementary results of the evolution of porosity and other parameters during dissolution for three-dimensional simulations and experiments can be found in Cha and Santamarina^73^ and Cha and Santamarina^47^.

**Figure 7:**
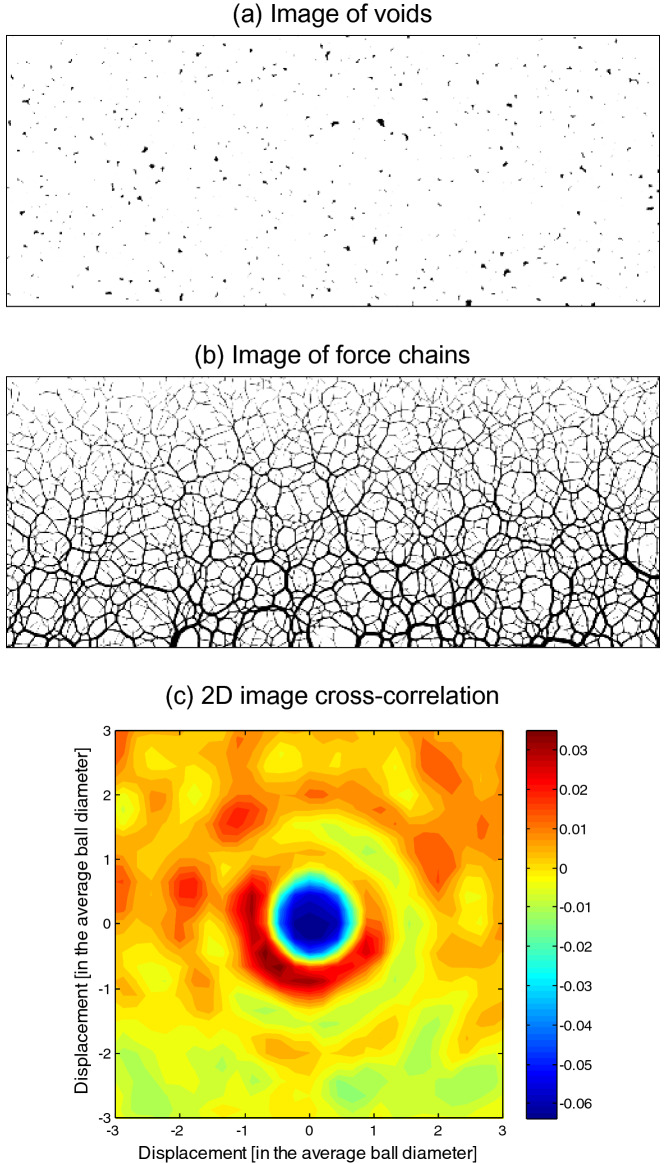
Causal link between (**a**) large remnant voids and (**b**) force chains; (**c**) 2D cross-correlation shows that large voids remain within a grain diameter from major force chains.

Careful inspection of the images in Fig. 6 reveals large remnant voids located next to major contact force chains after dissolution; this implies that force arches developed around the dissolving particles. A 2D cross-correlation analysis between the image of large voids (Fig. 7a; Note: Grains are grown ΔR/R≈50% without displacement to close all small voids) and an image of contact force chains (Fig. 7b) confirms that most large voids were found one particle diameter away from the major force chains (and typically below; see Fig. 7c).

This distinct post-dissolution internal fabric anticipates a different sediment response upon further loading, e.g., more prone to buckling under shear perturbation. In particular, the inherent shear loading in a sloping ground may trigger static liquefaction given the high contractive tendency in soils that experienced dissolution^47^. This may explain the shallow slide reported in Fig. 2b.

### Displacement

In agreement with the experimental results (Fig. 2a), vertical settlement was the prevailing global deformation pattern (Fig. S2 in the Supplemental Data). Sediments with higher soluble fractions and lower interlocking or macroscopic friction angles experienced larger amounts of settlement. Once again, vertical settlements increased quasilinearly toward the surface because of the random distribution of dissolvable grains. The third column in Table 2 shows vertical strains determined by surface settlements near the vertical walls (i.e., away from the slope). All vertical strains are well below 0.25, in agreement with increased porosity. Higher granular interlocking reduces vertical strain. In addition, vertical strains increase with the initial heights by a small degree due to gains in self-weight with increasing depth (Table 2).

**Table 2:** Normalized displacements—DEM simulations.

Initial slope angle	Hindered rotation (%)	*Vertical strain: Near vertical walls	^†^δ_v_/H_0_: Selection	^‡^δ_h_/H_0_: Selection
20°	0	0.212	0.215	0.235
	40	0.205	0.195	0.235
	80	0.163	0.130	0.065
30°	40	0.211	0.285	0.350
	80	0.176	0.175	0.122
40°	80	0.189	0.220	0.230

*: Heights of level-ground portion: β_0_ = 20°: 60 mm, β_0_ = 30°: 71.2 mm, β_0_ = 40°: 82 mm.

^†^: Vertical movements of grains at the top of the selection normalized by their initial heights.

^‡^: Horizontal movements of grains at the top of the selection normalized by their initial heights.

The sloping ground also creates significant mass wasting, even under the fully quasi-static dissolution. As the randomly distributed soluble particles dissolve and surrounding particles are rearranged, all of the particles are mobilized vertically and horizontally (see the video—Supplementary Data). Their mobilizations are coupled during dissolution in a way that as much as particles displace vertically, horizontal movements co-occur in the sloping ground, which bears large maximum shear stresses relative to mean normal stresses (Fig. 5).

**Figure 8:**
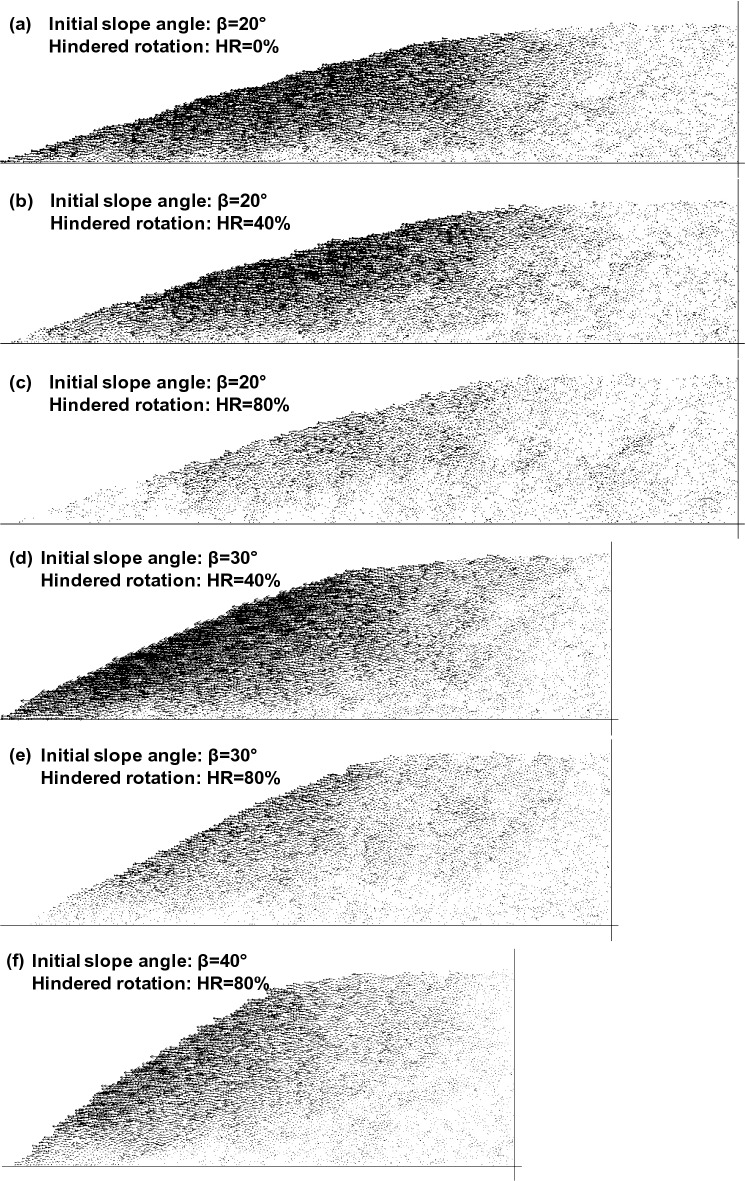
Horizontal component of the displacement vectors at the end of dissolution—All cases shown at the same displacement vector scale.

**Figure 9:**
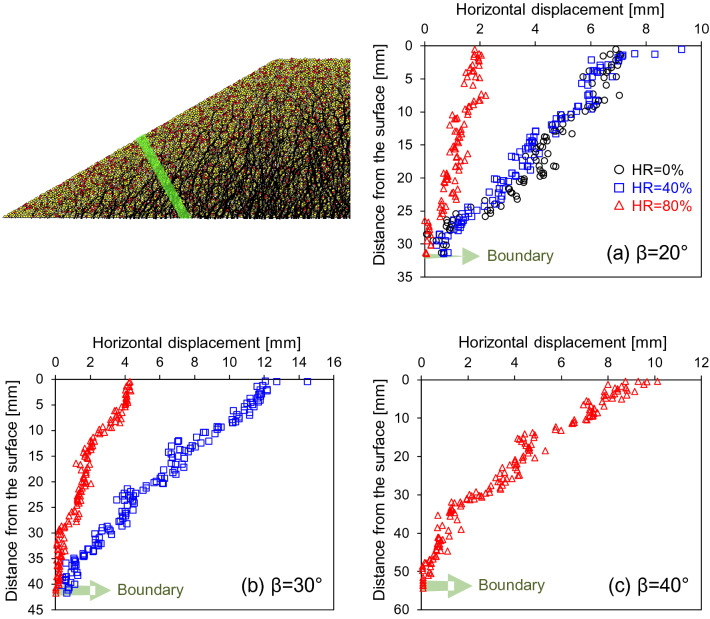
The horizontal granular displacements versus the initial distance perpendicular to the slope surface in the mid-slope. The green strip (4 mm width) shows the range of selected grains.

Figure 8 presents the horizontal components extracted from the final displacement vectors. Horizontal displacements were largest on the slope surface and decreased away from the slope surface without a sharp transition, that is, no shear localization. The horizontal displacements diminish gradually toward the level ground. Figure 9 quantifies the horizontal granular displacements versus the initial distance perpendicular to the slope surface in the mid slope. In all cases, horizontal grain displacement is maximum at the surface and decreases quasi-linearly with the distance from the slope surface, and becomes zero at the bottom boundaries, similar to the vertical granular displacement along the depth. It resembles simple shear flow although their movements are quasi-static.

**Figure 10:**
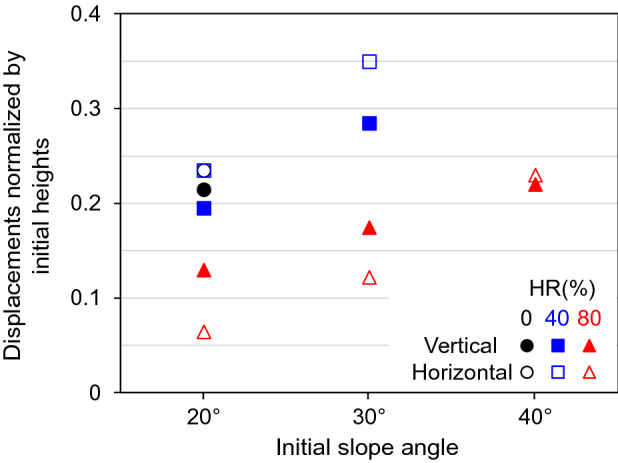
Vertical and horizontal granular displacements at the top of the selection (see Fig. 9) normalized by the initial heights from the base. Numerical data from Table 2 (the 4th and 5th columns).

Granular interlocking reduces horizontal displacement, and the larger initial slope angle promotes greater mass wasting (Figs. 8 and 9). Clearly horizontal displacements increase with the slope angles and decrease with granular interlocking more sensitively than the vertical displacements (Table 2 and Fig. 10). In addition, granular dissolution in sloping grounds increases vertical strain more than dissolution on the level ground (Table 2: compare the third and fourth columns); as grains displace horizontally, the slope geometry may create additional downward movement.

**Figure 11:**
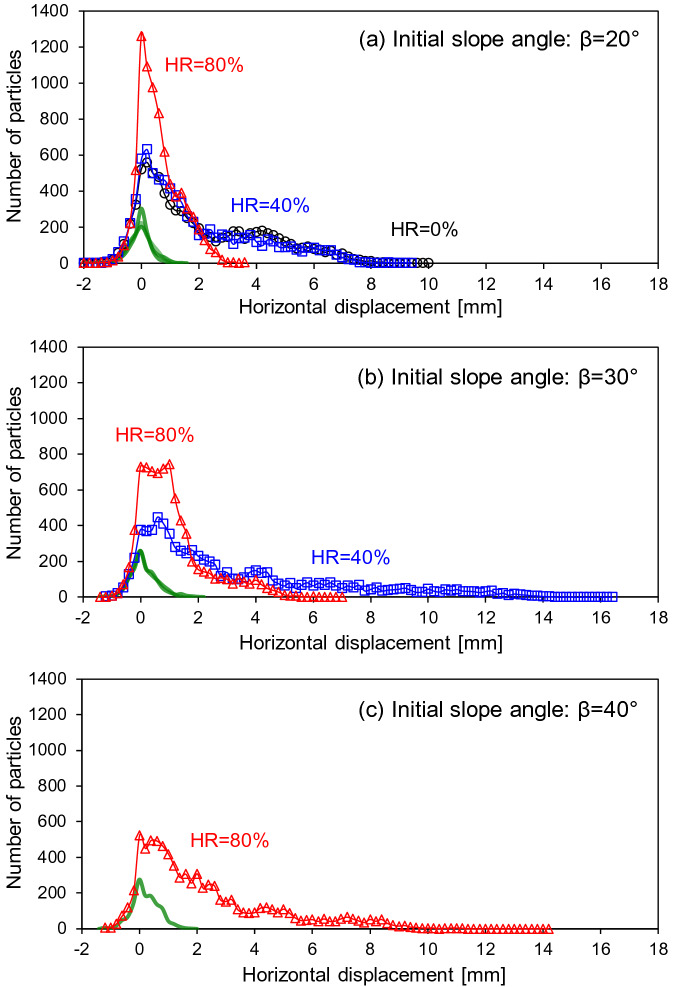
Histogram of horizontal displacements experienced by all non-dissolvable particles at the end of dissolution. Green lines show histograms for the ~ 2000 particles closest to the rigid wall on the right. Bin counts are connected to facilitate visualization and distinguish among HRs (Bin size = 0.2 mm for all cases).

Histograms of horizontal displacements after dissolution clearly indicate that the bulk of the medium had a mean horizontal displacement near zero (Fig. 11). The green lines show histograms for the ~ 2000 particles (19% of the total number of particles) in the level ground, closest to the rigid wall on the right. In this zone, the horizontal displacements of particles are small, contained within ± 2 mm without a preferential direction; the histogram is nearly symmetric about zero displacement (Fig. 11). When all of the particles are plotted, however, significant portions are displaced horizontally outward (positive direction) (Fig. 11), which represents the grain mass under the slope surface. Steeper slopes in sediments with lower granular interlocking or macroscopic friction angle showed higher horizontal displacements following dissolution, which is consistent with earlier results (Figs. 8, 9 and 10).

## Discussions

### Field implication: mass wasting due to granular mass loss

Certainly, the slope geometry and soluble distribution in this DEM study represent a particular case: a young sloping deposit with a limited slope extent such as energy waste or embankment that contains a soluble fraction overlies an insoluble base ground. Some field situations show that the slope surface and the lower end of the dissolution front may be parallel and the affected depth determined by the intensity and duration of each infiltration event. Rainwater infiltration may create secondary porosity in a young sloping deposit and weaken cementation in sloping sediments. In mass wasting events induced by freezing-thaw, the freezing and thawing depths are determined by the temperature difference. Layer(s) can also dominate a dissolution depth or geometry. Nevertheless, this study shows that horizontal/vertical grain movements are proportional to the affected depth of the dissolution. Also, the effects of major parameters are qualitatively recognized from this study and previous studies. While further, focused studies are required to accurately determine each effect to be useful for predicting the behavior, the following relation lends perspective to the combined effects in the absence of shear localization:

3 $${\updelta }_{h}, {\updelta }_{v}\propto L\cdot \frac{f(\theta )\cdot g(SF)\cdot j(D)}{k(\phi )}$$

where each function correlates positively with the parameter inside the parentheses. θ is the slope angle, SF the fraction of randomly distributed soluble particles, ϕ the macroscopic friction angle, L the dimension of a slope, either the height or length, and D (%) the progression of dissolution that can range from 0 to ~ 80%, after which the movement begins to stabilize.

### Final slope angles

**Figure 12:**
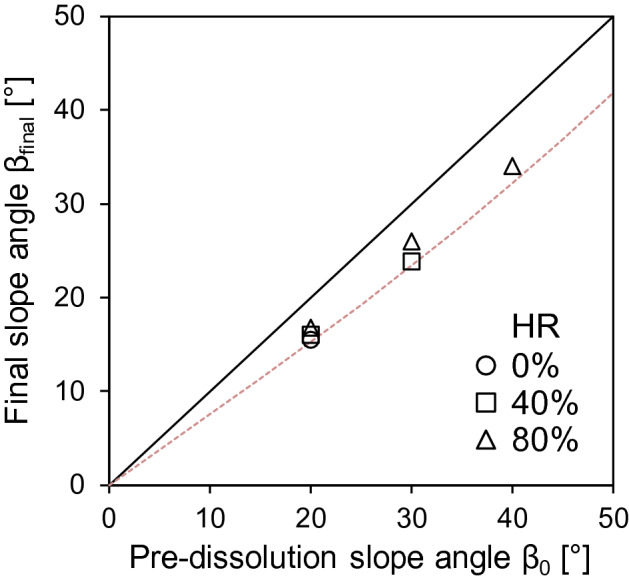
Comparison of the initial slope angle, β_o_, and the final slope angle after dissolution, β_final_, for all cases (SF = 25%). The dotted line is an upper bound estimate ΔH/H_o_ = SF when dissolution takes place at constant porosity.

The initial slope angle, β_0_, became flatter as dissolution progressed, and the final slope was β_final_ < β_0_ in all cases (Fig. 12). In the absence of undrained failure, lateral displacements during dissolution had a minor effect on the slope angle; in fact, a good approximation to the final slope angle was trigonometrically computed from the shortening, ΔH, of the initial land elevation, H_o_:

4 $$\tan \,\beta_{final} = \left( {1 - \frac{\Delta H}{{H_{o} }}} \right)\,\tan \,\beta_{o}$$

Apparently, the proximity of the initial slope angle, β_0_, to the numerically measured angle of repose, β_repose_, has a secondary effect; however, granular interlocking does reduce the vertical shortening (i.e., smaller ΔH/H_o_) and preserves steeper slopes after dissolution than less interlocked specimens, as predicted by the equation above. The soluble fraction, SF, is an upper bound estimate for vertical shortening, ΔH/H_o_ < SF—in fact, ΔH/H_o_ = SF when dissolution takes place at a constant porosity (dotted line in Fig. 12); otherwise, porosity increases during dissolution, as observed in this study.

The limited length of slope in this DEM study, as is often the case for embankments and engineered fills, creates a non-uniform slope movement. Horizontal granular displacement is more restricted near the base than on the mid slope because of the non-dissolving base material and its friction with grains of the sloping deposit. The horizontal displacement also diminishes gradually toward the level ground. Non-uniform horizontal movements thus result in slightly curved surfaces (more pronounced in high-angle slopes, e.g., Figs. 6f and 8f).

## Conclusions

Mineral dissolution and precipitation are concurrent soil processes and can take place in relatively short time scales in advective regimes and when minerals are exposed far from equilibrium, which is common in young natural and engineered systems. We investigated the effect of grain dissolution on slope stability using a 1 g model experiment complemented by discrete element simulations.

Upon dissolution, load-carrying grain arches develop around dissolving particles. A honeycomb-shaped contact force chain topology characterizes the high-porosity fabric after dissolution. The cross-correlation analysis confirms that large voids remain next to major contact force chains after dissolution. The sloping ground has high initial values of maximum shear stress relative to mean normal stress. After dissolution, the state of stress became more stable.

Dissolution leads to significant slope movements. While global vertical settlement is the prevailing deformation pattern, grains displace horizontally in the sloping ground, which leads to significant mass wasting even though the dissolution takes places quasi-statically. Horizontal granular movements are maximum at the slope surface and decrease linearly away from the slope surface bounded by the non-dissolving base, similar to vertical granular displacement along the depth. Sediments with a lower global friction angle or steeper slope experience larger displacements, both vertically and horizontally.

Slopes become flatter after dissolution; the reduction in slope angles directly related to the loss in ground elevation, ΔH/H_o_. Yet, for all cases, the slope angle is higher than the estimate from the upper bound for vertical shortening, ΔH/H_o_≈SF, because of increased porosities.

No shear localization or catastrophic failure was observed in the numerical simulations during dissolution under drained or dry conditions. However, sudden undrained shear may take place and lead to shallow failures. The sequence of events involves: (1) dissolution and increase in porosity, (2) internal stress approaching failure, (3) grain skeleton collapse, (4) pore pressure buildup, and (5) shear occurring as a form of static liquefaction.

## Supplementary Information

Supplementary Information 1.

Supplementary Information 2.

## Data Availability

The datasets used and/or analysed during the current study are available from the corresponding author on reasonable request.
