# Supplementary material for: Effect of grain dissolution on sloping ground
Source: Sci Rep. 2022 Dec 23;12:22203. doi: 10.1038/s41598-022-26620-1 (PMC9789098; doi:10.1038/s41598-022-26620-1)
Supplement: Supplementary file 1 — Supplementary Information 1. [file 41598_2022_26620_MOESM1_ESM.docx]

**Supplemental Data**

**Figure S1.** Rapid slide occurring during granular dissolution, captured by Particle Image Velocimetry using two consecutive images.

**
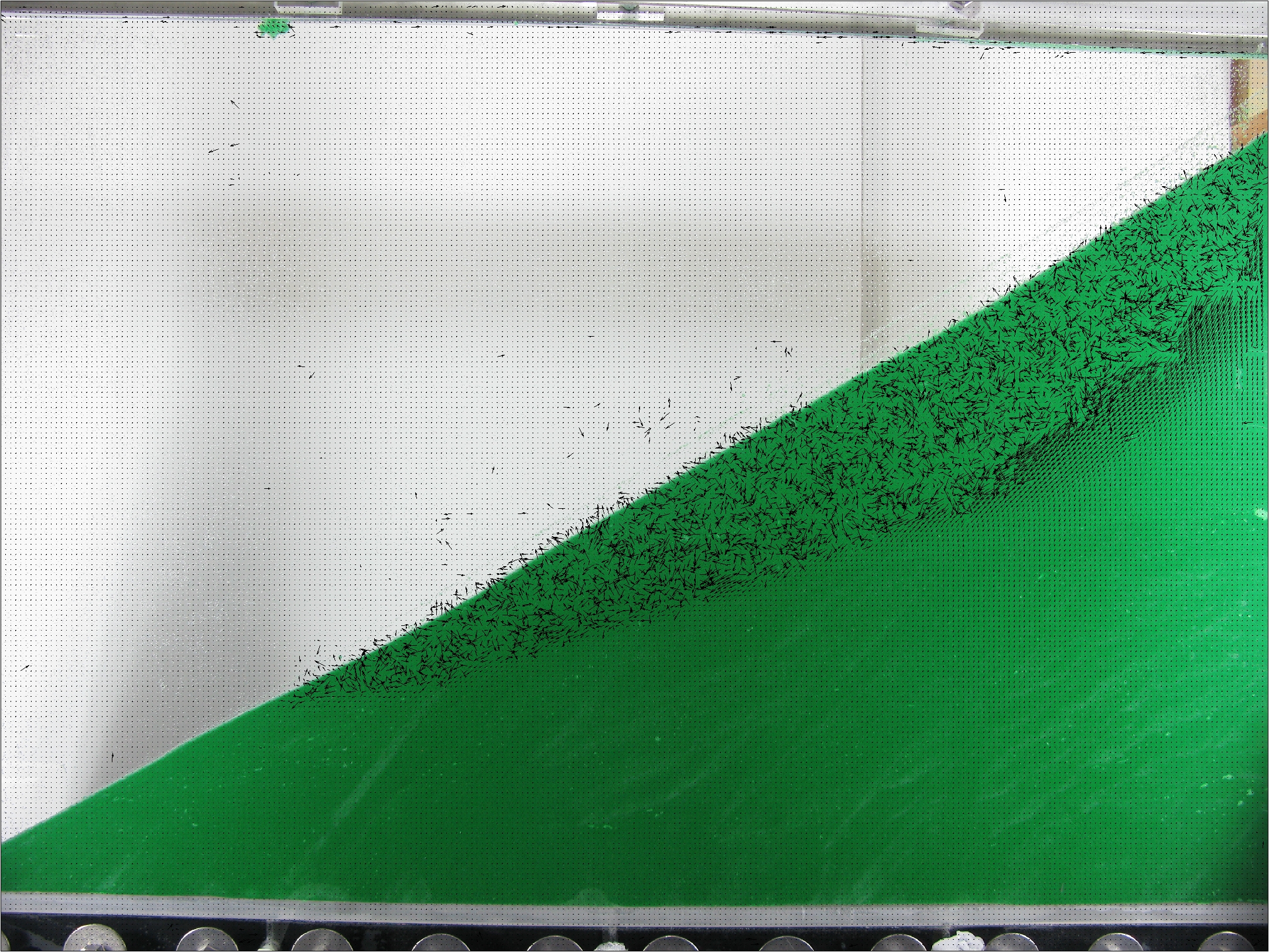
**

**Figure S2.** Displacement vectors at the end of dissolution - All cases shown at the same displacement vector scale.
