# Supplementary material for: Effect of grain dissolution on sloping ground
Source: Sci Rep. 2022 Dec 23;12:22203. doi: 10.1038/s41598-022-26620-1 (PMC9789098; doi:10.1038/s41598-022-26620-1)
Supplement: Supplementary file 2 — Supplementary Information 2. [file 41598_2022_26620_MOESM2_ESM.pptx]

## Slide 1
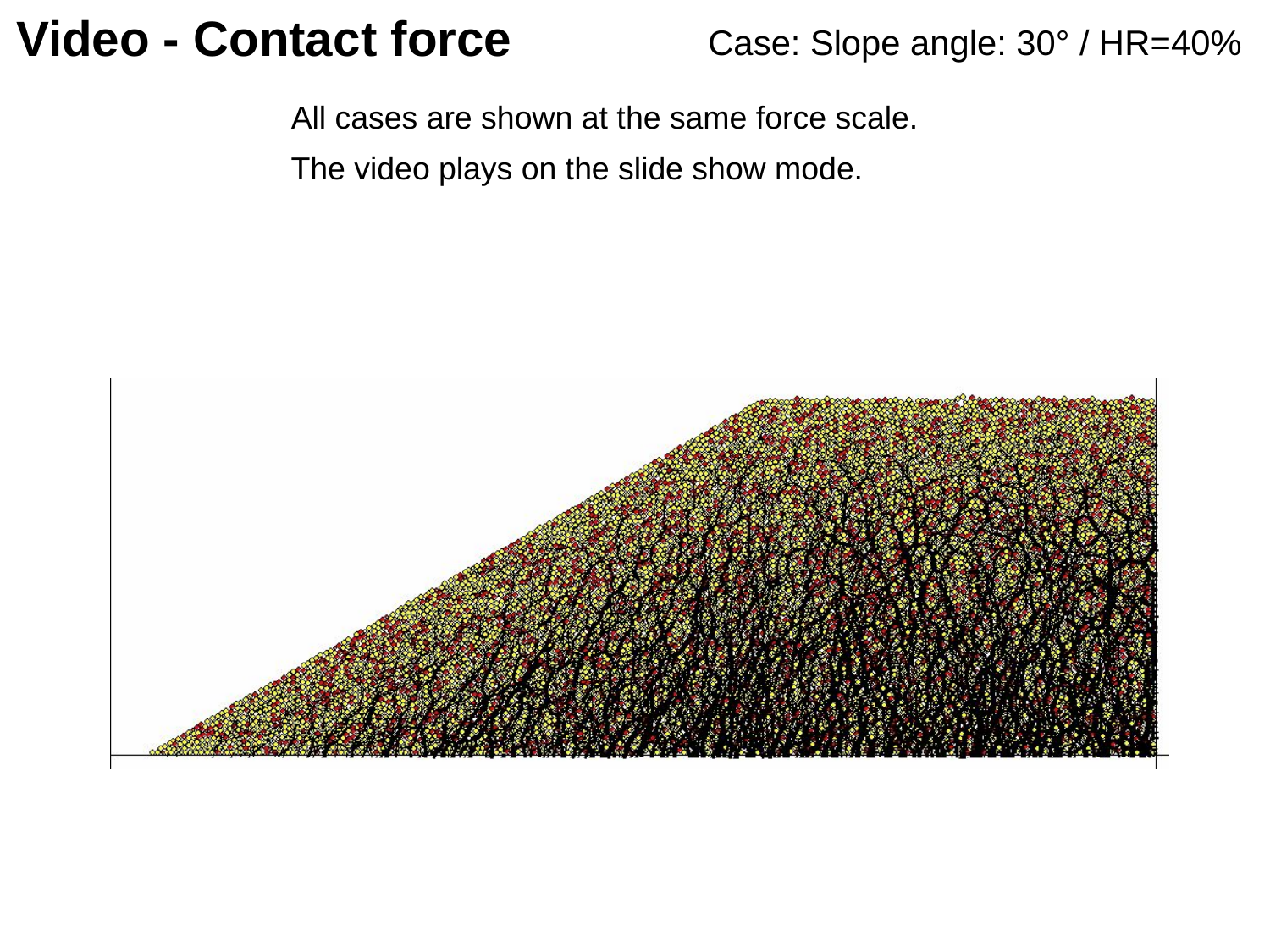

Video - Contact force
Case: Slope angle: 30° / HR=40%
All cases are shown at the same force scale.
The video plays on the slide show mode.

## Slide 2
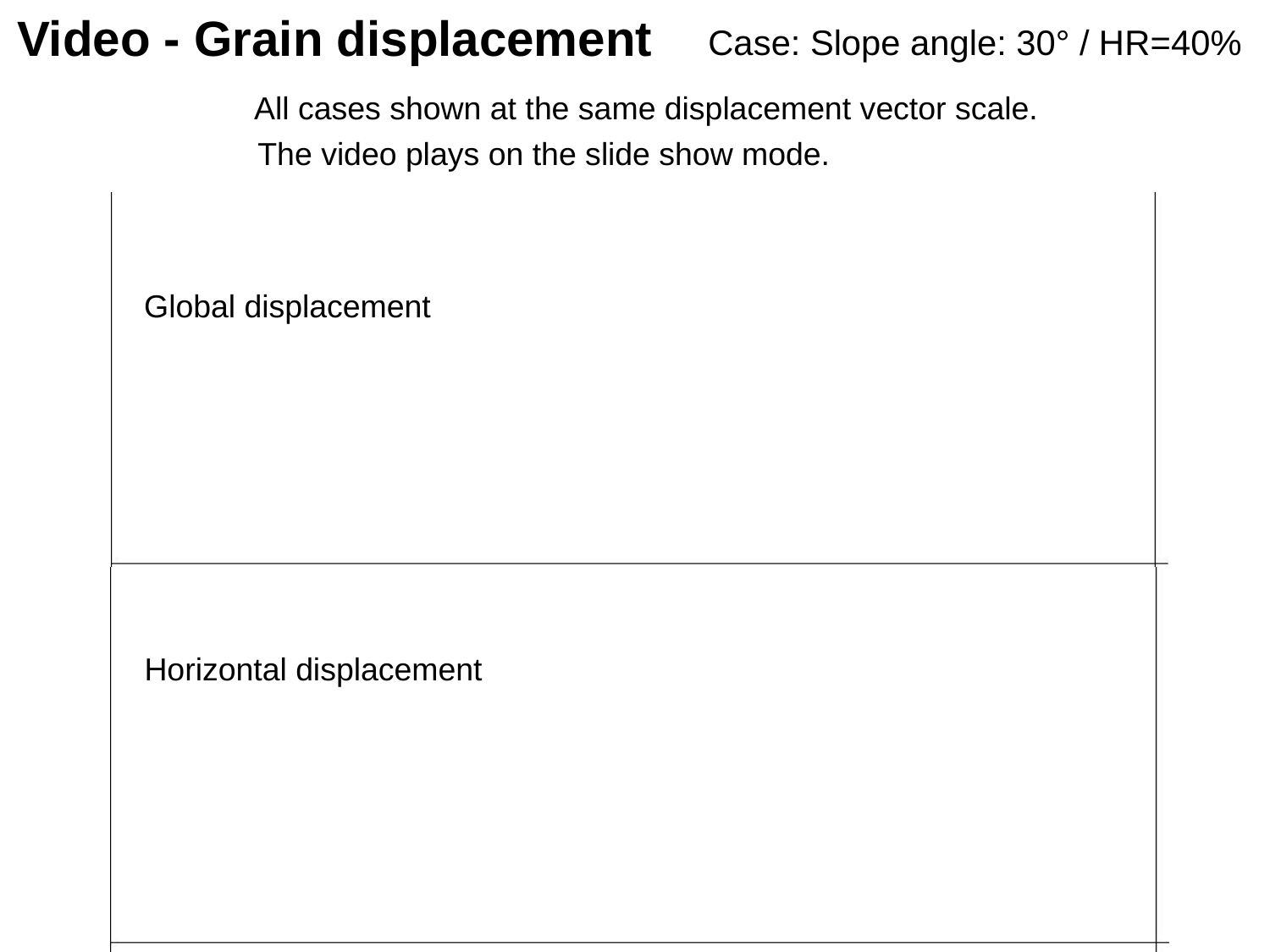

Video - Grain displacement
Case: Slope angle: 30° / HR=40%
All cases shown at the same displacement vector scale.
The video plays on the slide show mode.
Global displacement
Horizontal displacement
